# RDMAS: a web server for RNA deleterious mutation analysis

**DOI:** 10.1186/1471-2105-7-404

**Published:** 2006-09-06

**Authors:** Wenjie Shu, Xiaochen Bo, Rujia Liu, Dongsheng Zhao, Zhiqiang Zheng, Shengqi Wang

**Affiliations:** 1 Beijing Institute of Radiation Medicine, Beijing 100850, China; 2 Beijing Institute of Health Administration and Medicine Information, Beijing 100850, China; 3 College of Electro-Mechanic and Automation, National University of Defense Technology, Changsha, Hunan 410073, China; 4 Department of Computer Science and Technology, Tsinghua University, Beijing 100084, China

## Abstract

**Background:**

The diverse functions of ncRNAs critically depend on their structures. Mutations in ncRNAs disrupting the structures of functional sites are expected to be deleterious. RNA deleterious mutations have attracted wide attentions because some of them in cells result in serious disease, and some others in microbes influence their fitness.

**Results:**

The RDMAS web server we describe here is an online tool for evaluating structural deleteriousness of single nucleotide mutation in RNA genes. Several structure comparison methods have been integrated; sub-optimal structures predicted can be optionally involved to mitigate the uncertainty of secondary structure prediction. With a user-friendly interface, the web application is easy to use. Intuitive illustrations are provided along with the original computational results to facilitate quick analysis.

**Conclusion:**

RDMAS can be used to explore the structure alterations which cause mutations pathogenic, and to predict deleterious mutations which may help to determine the functionally critical regions. RDMAS is freely accessed via .

## Background

In addition to its central role in information transfer from DNA to protein, RNA performs a remarkable range of functions [1]. Large numbers of noncoding RNA (ncRNA) transcripts are being revealed [2]. Exploring the role and diversity of these numerous ncRNAs now constitutes a main challenge in life science [3]. In a broad sense, the list of functional ncRNAs also includes functional motifs within protein-coding genes, located mostly in the non-translated 5' or 3' regions of messenger RNAs.

Mutations in RNA genes may lead to striking alterations in RNA structures that impair functions, resulting in diseases. Mutations in some RNA regulators have been reported to be associated with neuropsychiatric disorders [4]. Mutations of tRNAs in mitochondria are reported to harbor more than half of all known mitochondrial pathogenic mutations [5]. Some recent researches also show that mutations in microRNA (miRNA) genes and its flanking sequences may contribute to cancer [6-8].

On the other hand, RNA deleterious mutations could be "beneficial" in some situation. The distribution of the recognized ribosomal functional sites and the antibiotic action sites has been found to be clearly correlated with the location of the known deleterious mutations in bacterial rRNAs. Therefore, deleterious mutations in rRNAs can serve as hallmarks of both functionally important ribosomal centers and antibiotic sites [9]. In their study on influenza viruses, Herlocher *et al*. found a nonsense mutation on PB2 segment which causes much difference in the secondary structure responsible for cold adaptation [10], that implies that viruses with similar deleterious mutations have potential for live vaccines.

In principle, a RNA mutation could be deleterious because it disrupts a functional site involved in catalysis, ligand-binding, or interaction with proteins. Since the functions of the ncRNAs critically depend on their specific structures, nucleotide alterations which result in structure change are expected to be deleterious. From this point of view, structure analysis should help to identify deleterious mutations. Some structure based method for RNA deleterious mutation analysis have been presented [11,12], which are applicable when few homologs are available. A user friendly Java application named RNAmute for RNA deleterious mutation analysis has also been reported [13,14].

The RDMAS we describe here is a noncommercial web application for RNA deleterious mutation analysis. Several secondary structure comparison methods have been implemented in RDMAS to evaluate structure deleteriousness of single nucleotide substitution in RNA molecules.

## Implementation

### Structural dissimilarity metric

**Figure 1:**
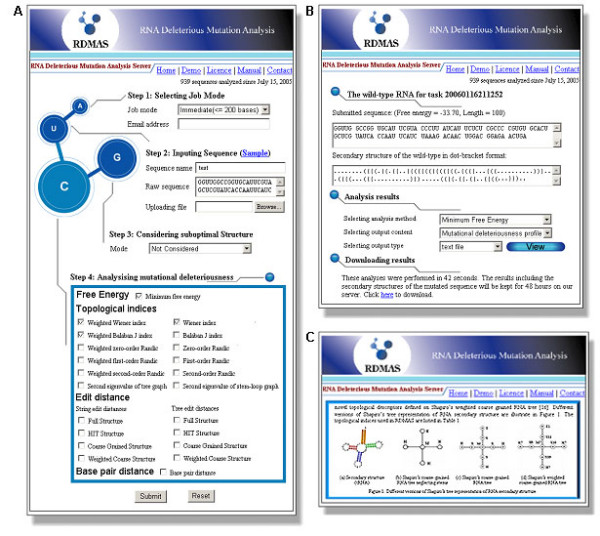
**Web interface of RDMAS**. (**A**) Input page. (**B**) Output page. (**C**) Online manual.

There are 3 × *N *possible single point mutations for a RNA molecule with *N *nucleotides. The deleteriousness of these mutations is analyzed in RDMAS on the basis of structure difference. The dissimilarity of secondary structures between wild-type and mutant, *D*(*R*, R^*^), is used to predict the deleteriousness of mutations. Four types of metric are employed, which are:

(i) Difference between free energy of RNA secondary structures, i.e.*D*(*R*, R^*^) = |*E*(*R*) - *E*(R^*^)|, where *E*(·) is the free energy computation function.

(ii) Edit distance between tree or their string representations of RNA secondary structures, i.e.*D*(*R*, R^*^) = *ED*(*R*, R^*^), where *ED*(·) represents the edit distance computation functions. The structure comparisons are implemented using Vienna RNA package [15,16] based on four different tree representations, including full, homeomorphically irreducible tree (HIT), coarse grained and weighted coarse representation.

(iii) Difference between topological indices of RNA structures, i.e. *D*(*R*, R^*^) = |*I*(*R*) - *I*(R^*^)|, where *I*(·) represents the topological index computation functions. Several topological indices defined on the RNA tree graph representation has been presented [12,17-20]. Suggested by Merris and tested by Barash's group, the Wiener index which has been widely used in computational biochemistry has also been introduced into RNA graph [21,22] recently. There is an interesting relation [23] between the Wiener number and the Laplacian spectrum of tree graph used in RNAMute. We have also proposed and employed novel topological descriptors defined on Shapiro's coarse grained and weighted coarse grained RNA tree [24] to characterize RNA structures (details will be published elsewhere). The topological indices used in RDMAS are listed in Table 1. Detailed descriptions can be found in the online manual of the web server (Figure 1C).

**Table 1:** Topological indices used to measure the structural difference between RNAs in RDMAS.

Topological index	Description
*λ*_*T*_	Second eigenvalue of Laplacian matrix of Shapiro's coarse grained RNA tree
λTw MathType@MTEF@5@5@+=feaafiart1ev1aaatCvAUfKttLearuWrP9MDH5MBPbIqV92AaeXatLxBI9gBaebbnrfifHhDYfgasaacH8akY=wiFfYdH8Gipec8Eeeu0xXdbba9frFj0=OqFfea0dXdd9vqai=hGuQ8kuc9pgc9s8qqaq=dirpe0xb9q8qiLsFr0=vr0=vr0dc8meaabaqaciaacaGaaeqabaqabeGadaaakeaaiiGacqWF7oaBdaqhaaWcbaGaemivaqfabaGaem4DaChaaaaa@313C@	Second eigenvalue of Laplacian matrix of Shapiro's weighted coarse grained RNA tree
^0^*χ*	Zero-order Randiæ index defined on Shapiro's coarse grained RNA tree
^1^*χ*	First-order Randiæ index defined on Shapiro's coarse grained RNA tree
^2^*χ*	Second-order Randiæ index defined on Shapiro's coarse grained RNA tree
^0^*χ*^*w*^	Zero-order Randiæ index defined on Shapiro's weighted coarse grained RNA tree
^1^*χ*^*w*^	First-order Randiæ index defined on Shapiro's weighted coarse grained RNA tree
^2^*χ*^*w*^	Second-order Randiæ index defined on Shapiro's weighted coarse grained RNA tree
*W*	Wiener index defined on Shapiro's coarse grained RNA tree
*W*^*w*^	Wiener index defined on Shapiro's weighted coarse grained RNA tree
*J*	Balaban index defined on Shapiro's coarse grained RNA tree
*J*^*w*^	Balaban index defined on Shapiro's weighted coarse grained RNA tree

(iv) Base pair distance between dot-bracket representations of RNA structures, i.e. *D*(*R*, R^*^) = *BP*(*R*, R^*^), where *BP*(·) represents the base pair distance computation function.

The secondary structure prediction in RDMAS is implemented using *RNAfold *and *RNAsubopt *[25] from the Vienna RNA package [15,16]. The former is a variation of the Zuker and Stiegler [26,27] minimum free energy problem that extends McCaskill's algorithm [28] and computes the complete density of states of an RNA sequence at predefined energy resolution, while the latter is for the calculation of all suboptimal structures within a user defined energy range above the MFE. In order to mitigate the uncertainty of the MFE structure, suboptimal structures of mutants within 1 kcal/mol (the default setting of *RNAsubopt*) above the minimum free energy (MFE) are considered. Three methods are used to estimate the difference between the structures of the wild-type and possible structure set of the mutant Γ^* ^= {R1∗
 MathType@MTEF@5@5@+=feaafiart1ev1aaatCvAUfKttLearuWrP9MDH5MBPbIqV92AaeXatLxBI9gBaebbnrfifHhDYfgasaacH8akY=wiFfYdH8Gipec8Eeeu0xXdbba9frFj0=OqFfea0dXdd9vqai=hGuQ8kuc9pgc9s8qqaq=dirpe0xb9q8qiLsFr0=vr0=vr0dc8meaabaqaciaacaGaaeqabaqabeGadaaakeaacqWGsbGudaqhaaWcbaGaeGymaedabaGaey4fIOcaaaaa@2FE5@, R2∗
 MathType@MTEF@5@5@+=feaafiart1ev1aaatCvAUfKttLearuWrP9MDH5MBPbIqV92AaeXatLxBI9gBaebbnrfifHhDYfgasaacH8akY=wiFfYdH8Gipec8Eeeu0xXdbba9frFj0=OqFfea0dXdd9vqai=hGuQ8kuc9pgc9s8qqaq=dirpe0xb9q8qiLsFr0=vr0=vr0dc8meaabaqaciaacaGaaeqabaqabeGadaaakeaacqWGsbGudaqhaaWcbaGaeGOmaidabaGaey4fIOcaaaaa@2FE7@,…, Rn∗
 MathType@MTEF@5@5@+=feaafiart1ev1aaatCvAUfKttLearuWrP9MDH5MBPbIqV92AaeXatLxBI9gBaebbnrfifHhDYfgasaacH8akY=wiFfYdH8Gipec8Eeeu0xXdbba9frFj0=OqFfea0dXdd9vqai=hGuQ8kuc9pgc9s8qqaq=dirpe0xb9q8qiLsFr0=vr0=vr0dc8meaabaqaciaacaGaaeqabaqabeGadaaakeaacqWGsbGudaqhaaWcbaGaemOBa4gabaGaey4fIOcaaaaa@305A@}, where Ri∗
 MathType@MTEF@5@5@+=feaafiart1ev1aaatCvAUfKttLearuWrP9MDH5MBPbIqV92AaeXatLxBI9gBaebbnrfifHhDYfgasaacH8akY=wiFfYdH8Gipec8Eeeu0xXdbba9frFj0=OqFfea0dXdd9vqai=hGuQ8kuc9pgc9s8qqaq=dirpe0xb9q8qiLsFr0=vr0=vr0dc8meaabaqaciaacaGaaeqabaqabeGadaaakeaacqWGsbGudaqhaaWcbaGaemyAaKgabaGaey4fIOcaaaaa@3050@ represents the *i *th predicted structure of the mutant. The two extreme values, *D'*(*R*, Γ^*^) = max⁡i{D(R,Ri∗)}
 MathType@MTEF@5@5@+=feaafiart1ev1aaatCvAUfKttLearuWrP9MDH5MBPbIqV92AaeXatLxBI9gBaebbnrfifHhDYfgasaacH8akY=wiFfYdH8Gipec8Eeeu0xXdbba9frFj0=OqFfea0dXdd9vqai=hGuQ8kuc9pgc9s8qqaq=dirpe0xb9q8qiLsFr0=vr0=vr0dc8meaabaqaciaacaGaaeqabaqabeGadaaakeaadaWfqaqaaiGbc2gaTjabcggaHjabcIha4bWcbaGaemyAaKgabeaakmaacmqabaGaemiraqKaeiikaGIaemOuaiLaeiilaWIaemOuai1aa0baaSqaaiabdMgaPbqaaiabgEHiQaaakiabcMcaPaGaay5Eaiaaw2haaaaa@3D20@ and *D'*(*R*, Γ^*^) = max⁡i{D(R,Ri∗)}
 MathType@MTEF@5@5@+=feaafiart1ev1aaatCvAUfKttLearuWrP9MDH5MBPbIqV92AaeXatLxBI9gBaebbnrfifHhDYfgasaacH8akY=wiFfYdH8Gipec8Eeeu0xXdbba9frFj0=OqFfea0dXdd9vqai=hGuQ8kuc9pgc9s8qqaq=dirpe0xb9q8qiLsFr0=vr0=vr0dc8meaabaqaciaacaGaaeqabaqabeGadaaakeaadaWfqaqaaiGbc2gaTjabcggaHjabcIha4bWcbaGaemyAaKgabeaakmaacmqabaGaemiraqKaeiikaGIaemOuaiLaeiilaWIaemOuai1aa0baaSqaaiabdMgaPbqaaiabgEHiQaaakiabcMcaPaGaay5Eaiaaw2haaaaa@3D20@ are taken for the most optimistic and the most pessimistic estimation, respectively. The synthetic estimation is given by summing the contribution of all structures weighted by their Boltzmann probabilities, which is similar to the methods used in some research [29]. In this case, the deleteriousness is given by D′(R,Γ∗)=∑i=1nwi⋅D(R,Ri∗)/∑i=1nwi
 MathType@MTEF@5@5@+=feaafiart1ev1aaatCvAUfKttLearuWrP9MDH5MBPbIqV92AaeXatLxBI9gBaebbnrfifHhDYfgasaacH8akY=wiFfYdH8Gipec8Eeeu0xXdbba9frFj0=OqFfea0dXdd9vqai=hGuQ8kuc9pgc9s8qqaq=dirpe0xb9q8qiLsFr0=vr0=vr0dc8meaabaqaciaacaGaaeqabaqabeGadaaakeaadaWcgaqaaiqbdseaezaafaGaeiikaGIaemOuaiLaeiilaWIaeu4KdC0aaWbaaSqabeaacqGHxiIkaaGccqGGPaqkcqGH9aqpdaaeWbqaaiabdEha3naaBaaaleaacqWGPbqAaeqaaaqaaiabdMgaPjabg2da9iabigdaXaqaaiabd6gaUbqdcqGHris5aOGaeyyXICTaemiraqKaeiikaGIaemOuaiLaeiilaWIaemOuai1aa0baaSqaaiabdMgaPbqaaiabgEHiQaaakiabcMcaPaqaamaaqahabaGaem4DaC3aaSbaaSqaaiabdMgaPbqabaaabaGaemyAaKMaeyypa0JaeGymaedabaGaemOBa4ganiabggHiLdaaaaaa@53DA@, where wi=exp⁡{−[E(Ri∗)−E(RMFE∗)]/kT}
 MathType@MTEF@5@5@+=feaafiart1ev1aaatCvAUfKttLearuWrP9MDH5MBPbIqV92AaeXatLxBI9gBaebbnrfifHhDYfgasaacH8akY=wiFfYdH8Gipec8Eeeu0xXdbba9frFj0=OqFfea0dXdd9vqai=hGuQ8kuc9pgc9s8qqaq=dirpe0xb9q8qiLsFr0=vr0=vr0dc8meaabaqaciaacaGaaeqabaqabeGadaaakeaacqWG3bWDdaWgaaWcbaGaemyAaKgabeaakiabg2da9iGbcwgaLjabcIha4jabcchaWnaacmqabaWaaSGbaeaacqGHsislcqGGBbWwcqWGfbqrcqGGOaakcqWGsbGudaqhaaWcbaGaemyAaKgabaGaey4fIOcaaOGaeiykaKIaeyOeI0IaemyrauKaeiikaGIaemOuai1aa0baaSqaaiabd2eanjabdAeagjabdweafbqaaiabgEHiQaaakiabcMcaPiabc2faDbqaaiabdUgaRjabdsfaubaaaiaawUhacaGL9baaaaa@4CF6@.

### Input and options

With a step-by-step style input interface (Figure 1A), the RDMAS web server is easy to use. The sequence of a RNA molecule can be input either by pasting raw sequence or by uploading sequence file in FASTA format. Multi-FASTA (MFA) format sequence file is also supported to facilitate users. The limit of sequence length is 200 bases for immediate jobs and 2,000 bases for batch jobs, which meets the need of ncRNA analysis in most cases. For batch jobs, a valid email address is required. The analysis scheme is designed to be custom-built for users. The algorithms for computing structure difference and the methods for using the sub-optimal structures can be selected by users.

### Output

The intermediate result report page will be refreshed automatically every 5 seconds after immediate jobs submission. The output page (Figure 1B) of an immediate job can be seen within 1 minute. Served as an online interactive analysis interface, all the output result can be viewed as graphic representation or text list by selecting the content item and clicking the "view" button on the output page. For batch jobs, a notification email containing a URL linked to the output page will be sent to the user when the job has been completed. The URL remains valid for 48 hours.

**Figure 2:**
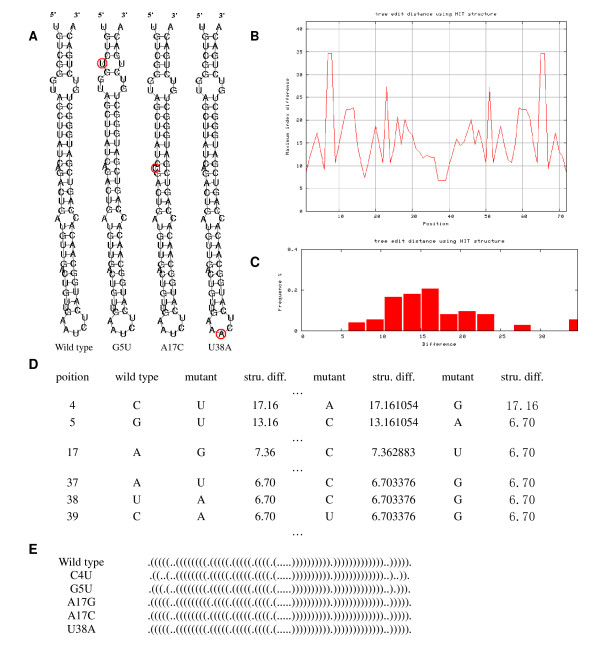
**Analysis result of microRNA miR-21 precursor**. The computation is based on the weighted second-order Randiæ index ^2^*χ*^*w *^defined on the stem-loop graph representation. (A) Structure illustrations of the wild-type and two mutants with great deleteriousness (G5U, A17C and U38A). The mutated nucleotides are marked by red circles. (B) Structural deleteriousness profile of pre-miR-21. (C) Deleteriousness distribution histogram of pre-miR-21. (D) Structural deleteriousness of some possible mutants of pre-miR-21. (E) Secondary structures of some possible mutants of pre-miR-21.

To make the analysis results intuitive, the maximum difference in structures between the wild-type and the possible mutants at each position are extracted into a structural deleteriousness profile and plotted as waveforms (Figure 2B). The structurally important sites can be easily revealed by peaks with high structural deleteriousness on the profile. The list of the structural deleteriousness values (Figure 2D) and the corresponding dot-bracket representations of secondary structures (Figure 2E) can be displayed as plain text on the output page.

The statistical distributions of the deleteriousness value are calculated and illustrated as histograms (Figure 2C), which may facilitate the analysis on RNA mutational robustness.

With a hyperlink located at the bottom of the output page (Figure 1B), the output page offers download of the results as a single packed file in ".gz" format for off-line analysis. In addition to the structural deleteriousness profile and deleteriousness distribution histogram (all in "PNG" image format), the secondary structure illustration of the wild-type and the mutants (all in PostScript format) are also included in the result file. The result file name is in the form "yymmddhhmmss.no", where "yy" is year, "mm" is month, "dd" is day, "hh" is hour, mm is minute, "ss" is second and "no" is serial number.

## Results and discussion

### Performance of the web server

**Figure 3:**
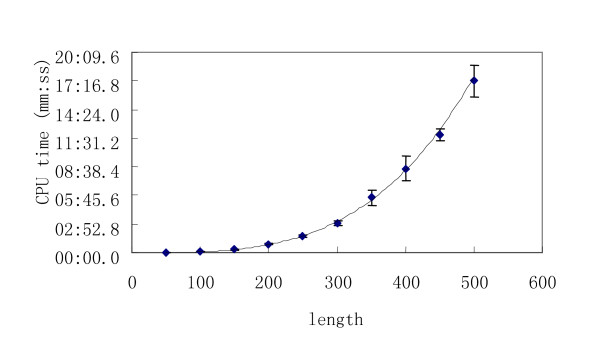
The CPU time of 10 groups of tests.

To test the computational efficiency of RDMAS, 500 random sequences (listed in Additional files) with 10 different lengths were submitted. All types of structure distance measurement are used in these tests. The CUP time of these 500 tests is illustrated in Figure 3.

### Case study

By using artificial mutants, some investigations have been done on the sequence and structural requirements for miRNA processing and functions [30-32]. These experimental results have shown that the base-pairing at the base of the precursor stem is critical for miRNA processing, while the internal loops, terminal loops and bulges are proved to be not essential.

To demonstrate how our web application can be helpful to the analysis on deleterious mutations in ncRNAs, the precursor of human miRNA miR-21 (pre-miR-21), a stem-loop of 71 nt, has been analyzed using RDMAS. Figure 2B is the structural deleteriousness profile of pre-miR-21 computed based on the tree edit distance of HIT representation. Figure 2C is the corresponding deleteriousness distribution histogram. The structures of the wild-type and three mutants G5U, A17C and U38A are illustrated in Figure 2A. The structural deleteriousness of possible mutants and the corresponding dot-bracket representations of the structures are listed partly in Figure 2D and Figure 2E.

It is shown that most mutants in pre-miR-21 are not deleterious. The mutations opening the base of the precursor stem lead to marked difference in RNA structure, while the mutations in the terminal loop and bulge seem to be less deleterious. These results are in good accord with the main conclusions drawn in the aforementioned experimental studies.

### Future works

Although the suboptimal structures of the mutant can be used in RDMAS, the structural distance measurement using multiple predicted structures is still a challenge to the present methods. Further research is needed to find approaches to measure the structural distance taking suboptimal structures of both the wild-type and the mutant into consideration at the same time.

On the basis of the criteria of conservation and compensatory co-evolution, Kondrashov presented a method using multiple homologous sequences to predict pathogenic mutations in mitochondria encoded human tRNAs [33]. In some other mutation studies on ncRNAs, especially on viral and bacterial RNAs, enough amounts of homologous sequences are also available. Our further research will also focus on developing methods for RNA deleterious mutation analysis using both homologic and structural information.

## Conclusion

Compared to single nucleotide mutation analysis in protein-coding gene, research on RNA mutation has been insufficient, both bioinformatics algorithms and applications are needed. Like RNAmute [13], the RDMAS we developed is a non-commercial software for RNA deleterious mutation analysis, and will be helpful both in the researches on the structure-function relationship of ncRNAs (such as functionally critical region identification) and in the RNA-targeted drug design.

## Availability and requirements

**Project name**: RDMAS

**Project home page**: 

**Operating system(s)**: Linux, Unix (no GUI)

**Programming language**: C and PHP

**Other requirements**: Vienna RNA package

**License**: GPL

**Restrictions to use by non-academics**: on request

## Authors' contributions

WS and XB designed and developed the methodology. WS and RL programmed the web application. DZ and ZZ test the software. XB wrote the manuscript. SW guided the project.
